# Supplementary material for: Genome-Wide Identification and Analysis of the Heat-Shock Protein Gene in L. edodes and Expression Pattern Analysis under Heat Shock
Source: Curr Issues Mol Biol. 2023 Jan 9;45(1):614–27. doi: 10.3390/cimb45010041 (PMC9858218; doi:10.3390/cimb45010041)
Supplement: Supplementary file 1 [file cimb-45-00041-s001.zip › cimb-2099158-supplementary.pdf]

Table S1. Primer pairs used for gene expression

Target gene	Description	Forward	Reverse	Length(bp)	Tm(℃)
LeHSP100. 1	Target gene	CATCGTCAAGCGGGCACT	TGGGCAATGTAAGAATCGTG	150	84
LeHSP100. 2/3	Target gene	GTCTGCTATAATGGCTGGTT	CTTCGTCTTCGATGTCCC	83	81
LeHSP40. 1	Target gene	GTGAAGTTGAACGTCGTGTTT	TTGCATCGTCCTGATATTCC	178	86. 1
LeHSP40. 2	Target gene	CCAATAACTGGCACAGGGAC	GGAACACGCACATCAACATC	182	85. 6
LeHSP40. 4	Target gene	TCGCTGCTAAGGACCTGTAC	GTCGGGATGATATTTCCGGCTCAGT	104	85. 6
LeHSP60. 3	Target gene	ATGCTCCGACATTACCAG	AGACTTCGCTCTACTTCCTC	168	84. 3
LeHSP60. 4	Target gene	GCCATGATCGCTCGTACTGCT	TTGCCAAGTCAAATCCTTCAGC	157	84. 9
LeHSP60. 6	Target gene	AGGGCGAATCCCAAACAA	AAGCACCAGATCACATCCAACT	128	83. 3
LeHSP70. 2	Target gene	AGTCACTTTCCCTGTCTACGA	GACCTTCAGCAGACCGTTAG	154	84. 3
LeHSP70. 3	Target gene	GCGTACCACTGACCCAT	CCCTGAATAGCAGCACC	195	86. 4
LeHSP70. 4	Target gene	TCCTCGCTCGGAACCTA	GCACCCTGAACCCTTTT	120	82. 8
LeHSP90. 1	Target gene	TACTCCGCATACTTGGTTG	GAGGGTTGACAGTGTCCTG	124	82. 6
LeHSP90. 2	Target gene	CGGTGTATCGTTCAAAGC	CCTCCGCATCTACCACTA	188	83. 9
LeSHSP. 1	Target gene	ACACTAACCAACCGTCCTATT	GGCCAAGTCACTATCAATATGCCCT	205	85
LeSHSP. 4	Target gene	GCGGTGAAAGTCAAGGTC	TGCGGCAACAGTCAAACG	191	84
LeSHSP. 5	Target gene	GCCTTCTTACCACTTTGACC	CACTGTACTAGCAGCGTCTCC	109	83. 5
LeTUB	Reference gene	GACATTTGCTTCCGAACCCT	CGGACATAACAAGGGACACA	79	80. 7
LeUBI	Reference gene	CGTCGGGTTCTACGAGAAAG	GTTCCGTGCTTTGTTCCTTG	133	82

Table S2. Information of Domains in HSPs.

HSPs	Domain number	Domain name	Location	
LeSHSP.1	1	ACD_sHsps-like	71	175
LeSHSP.2	1	IbpA	50	154
LeSHSP.3	1	ACD_sHsps-like	50	185
LeSHSP.4	1	IbpA	53	155
LeSHSP.5	1	IbpA	52	155
LeSHSP.6	1	IbpA	50	153
LeSHSP.7	1	IbpA	43	146
LeSHSP.8	1	ACD_sHsps-like	151	229
LeHSP40.1	2	DnaJ_C	202	333
		DnaJ	7	64
LeHSP40.2	2	DnaJ_C	292	423
		DnaJ	74	135
LeHSP40.3	2	DnaJ_C	209	343
		DnaJ	6	68
LeHSP40.4	2	DnaJ_C	198	344
		DnaJ	20	81
LeHSP40.5	2	DnaJ_C	225	356
		DnaJ	4	66
LeHSP40.6	2	DnaJ_C	276	412
		DnaJ	12	63
LeHSP60.1	1	Cpn60_TCP1	44	534
LeHSP60.2	1	Cpn60_TCP1	33	539
LeHSP60.3	1	Cpn60_TCP1	37	528
LeHSP60.4	1	Cpn60_TCP1	30	541
LeHSP60.5	1	Cpn60_TCP1	49	573
LeHSP60.6	1	Cpn60_TCP1	32	555
LeHSP60.7	1	Cpn60_TCP1	45	538
LeHSP60.8	1	Cpn60_TCP1	38	543
LeHSP60.9	1	Cpn60_TCP1	29	516
LeHSP70.1	1	HSP70	6	611
LeHSP70.2	1	HSP70	9	607
LeHSP70.3	1	HSP70	39	604
LeHSP70.4	1	HSP70 superfamily	25	724
LeHSP70.5	1	HSP70	50	654
LeHSP70.6	1	HSP70 superfamily	22	512
LeHSP90.1	2	HSP90	183	673
LeHSP90.1		ATPase_c	26	181
LeHSP90.2	2	HSP90	223	768
LeHSP90.2		ATPase_c	54	163
LeHSP100.1	5	AAA_2	603	767
		AAA_lid_9	346	446

		ClpB_D2-small	773	852
		AAA	204	341
		Clp_N	95	145
			22	67
LeHSP100.2	4	AAA_2	478	645
		AAA_lid_9	219	322
		ClpB_D2-small	653	731
		AAA	82	211
LeHSP100.3	4	AAA_2	464	631
		AAA_lid_9	205	308
		ClpB_D2-small	639	717
		AAA	68	197

Table S3. Amino sequence of motifs in HSPs

Motifs	Amino acid sequence
Motif 1	MSLYFYEPSYQWDRFFDRAFGSAASRGGZ
Motif 2	GQSQALTERSD
Motif 3	FIRPKMDLHEDKEKNLVTATFELPGVKKEDIQLDVHBGRLT
Motif 4	KVSEEHEZDGYAVRERSYGKFSRTLQLPRGVKEE
Motif 5	EIKASMEBGVLTVTFPKATPEEKPKKITI
Motif 6	TDYYELLGVSPDASEDDIKKAYKKLALKYHPDKNAD
Motif 7	FKEIAEAYEILSDEEKRAVYD
Motif 8	VTLEDLYKGKTVKLKITRRVLCGSCKGSG
Motif 9	AGDVVFVVEEAPHDRFKRQGNDLLVDVKIPLLEAL
Motif 10	GDLFVKFNVVLP
Motif 11	SNIVAALALABVVRTSLGPRGMDKMJVDAKGE
Motif 12	TNDGATILKEIZVEHPAAKLLVDJSQAQDDEVGDGTTSVVVLAGELL
Motif 13	TILLRGSNEQIVDEVERALHDALSVVKNLVKDGRLVPGGGA
Motif 14	KGKEQYAVQAFASALEVIPRTLAENAGLDSID
Motif 15	GGIVDMTEAGVWEPLLVKRQAJKSATEAAVLLLRVDDIVQA
Motif 16	INSFYSNKEIFLRELISNANDA
Motif 17	IRDTGIGMGKEELVNNLGTJAKSGTKGFL
Motif 18	LIGQFGLGFYSAFLVAEKVQVISKPNDDEKYPW
Motif 19	WEELNGQKPIWTRDPKEITDEEYSAFYKAFFKDWEDPLSWKHFS
Motif 20	VVISNRJVDSPCVJVAGQFGWSANMZRIM
Motif 21	EEIRRTIQILSRRTKSNPVLIGPPGVGKTAILEGLASRIVAKEVPESLQN
Motif 22	TTPDEYRKTIGKDAALERRFQPVSIDEPTVESTISILRGLKPRYEVHHGV
Motif 23	VVGQDHVVTAISDAVRMSRAGLQAPNRPVASFLFLGPTGVGKTELCKALA
Motif 24	FNDEQRGLININMSEYHDRHTISRLIGAAPGYVGFEEGGQLTEAVRRKPY
Motif 25	HKDVAMILLQILDEGSVTDSQGRKVDFKNTIICLTSNLGSDILAHKSDCN
Motif 26	IGIDLGTTYSCVGVWQGDRVEIIANDQGNRTTPSYVAF
Motif 27	ERLIGDPAKNQAAMNPKNTVFDAKRLIGRKFDDQEVQKDMKHWPFEVVEK
Motif 28	FLGKKVTHAVITVPAYFNDAQRQATKDAGTIAGLDVLRIINEPTAAAIAY
Motif 29	TRARFEELVEDLFKSTLDPVEKVLKDAKVKKDKIDEIVLVGGSTRIPKIQ
Motif 30	YEGERTQTKDNNLLGKFELSGIPPAPRGVPQIEVTFDIDANGILKVSAAD
